# Alterations in Gene Expression and Alternative Splicing Induced by Plasmid-Mediated Overexpression of GFP and *P2RY12* Within the A549 Cell Line

**DOI:** 10.3390/ijms26072973

**Published:** 2025-03-25

**Authors:** Qingqing Liu, Zhaoyu Liu, Yongqi Qian, Mingxu Wu, Jing Mo, Can Wang, Guoqing Xu, Liang Leng, Sanyin Zhang

**Affiliations:** 1 College of Basic Medical Sciences, Chengdu University of Traditional Chinese Medicine, Chengdu 611137, China; qingqingliue@163.com (Q.L.); qianyq0218@163.com (Y.Q.); 15208107547@163.com (M.W.); 2 Institute of Herbgenomics, Chengdu University of Traditional Chinese Medicine, Chengdu 611137, China; liuzhaoyu2008@hotmail.com (Z.L.); jingmoe@163.com (J.M.); wangcan@cdutcm.edu.cn (C.W.); xuguoqing0201@163.com (G.X.); 3 School of Chinese Materia Medica, Tianjin University of Traditional Chinese Medicine, Tianjin 300193, China; 4 Innovative Institute of Chinese Medicine and Pharmacy, Chengdu University of Traditional Chinese Medicine, Chengdu 611137, China

**Keywords:** overexpression cell line, DEGs, gDTUs, A549, GFP, *P2RY12*

## Abstract

Phenotypic modifications and their effects on cellular functions through the up-regulation of target gene expression have frequently been observed in genetic studies, but the unique roles of cell lines and their introduced plasmids in influencing these functions have not been fully revealed. In this research, we developed two distinct cell lines derived from the A549 cell line: one that stably overexpresses GFP and another that is a polyclonal stable line overexpressing both GFP and *P2RY12*. We then utilized transcriptome sequencing (RNA-seq) technology to screen out differentially expressed genes (DEGs) and genes with differential transcript usage (gDTUs) after GFP overexpression (GFP-OE) and *P2RY12* overexpression (*P2RY12*-OE). We found that, compared with A549, there were more than 1700 differentially expressed genes (DEGs) in both GFP-OE and *P2RY12*-OE cells, while only 866 DEGs were identified in GFP-OE and *P2RY12*-OE cells. Notably, the differences in transcript usage were relatively minor, with only over 400 genes exhibiting changes across all three groups. The functional analysis of DEGs and gDTUs showed that they were both highly enriched in the pathways associated with cell proliferation and migration. In summary, we performed an extensive analysis of the transcriptome profile of gene expression and alternative splicing with GFP-OE and *P2RY12*-OE, enhancing our comprehension of how genes function within cells and the processes that control gene expression.

## 1. Introduction

In 1952, Joshua Lederberg initiated the classification of genetic elements found in the cytoplasm by introducing the term “plasmid”, which is “a generic term for any extrachromosomal genetic determinant” [1]. Plasmids are circular forms of DNA that can replicate on their own, separate from the chromosomal DNA of the host, and are mainly located in bacterial cells [2]. The characteristics of plasmids, including adaptability, compatibility, safety, and cost-effectiveness, have enabled molecular biologists to utilize them for a range of applications, including gene expression, cloning, amplification, and gene delivery [3,4]. Researchers frequently employ plasmids in studies of gene expression by inserting the coding sequences of specific genes into plasmids or viral vectors. They take advantage of the regulatory components present in the vector’s backbone to facilitate high levels of transcription and translation of these genes, which is crucial to understanding gene functions and their regulatory mechanisms [5]. It has been demonstrated in multiple studies that the transcription levels of cells are influenced by factors such as the length of the plasmid insert, the copy number of the plasmid, its structural design, the presence of different sequences, and the efficiency of transfection [6,7]. The influences pertain to several levels of regulating gene expression, including transcription, translation processes, and the alternative splicing of mRNA. The level of gene expression after plasmid transfection is determined by the interplay of these various factors [8,9].

*P2RY12*, a GPCR activated by adenosine diphosphate, is present in low quantities in lung adenocarcinoma (LUAD) tissues. Its increased expression has been shown to reduce the proliferation and migration of A549 cells, suggesting it may be a valuable target for therapy and a potential prognostic marker [10]. GFP plasmids can create fusion proteins with specific focal genes, and these fusion proteins continue to exhibit fluorescence excitation properties [11]. They are frequently utilized as controls for labeling and monitoring cells to assess their transfection success [12]. Typically, GFP functions as a reporter gene without having a direct impact on the biological functions of cells. Nevertheless, studies have demonstrated that the presence of GFP-tagged fusion proteins can be detrimental to the nucleus, which might impact gene expression, especially when these proteins are expressed at elevated levels or for prolonged periods [13,14]. Furthermore, the simultaneous expression of GFP alongside other genes can influence cell growth characteristics by modifying the activity of these genes. In certain instances, the use of GFP-tagged expression systems can influence cell growth indirectly, which varies based on the gene or protein being co-expressed [15]. Therefore, to understand the influence of GFP on distinct cell functions such as proliferation, it is crucial to analyze it in conjunction with specific experimental contexts and co-expressed genes.

The process of high-throughput screening in drug discovery today typically begins with the employment of cell libraries, such as those that encompass genome-wide pan-GPCR cells for GPCR-focused drug research [16,17]. To explore how plasmid transfection influences gene expression and alternative splicing, we conducted RNA sequencing on the transcriptomes regulated by *P2RY12* and GFP in human A549 cells, utilizing the target gene plasmid *P2RY12* alongside its control plasmid, GFP. A comparative analysis of transcriptomes showed that the elevated expression of GFP and *P2RY12* influenced gene expression dynamics and alternative splicing processes within the cells. Moreover, the overexpression of GFP and *P2RY12* might play a role in regulating gene expression and alternative splicing concerning apoptosis and cell proliferation, which serves as an experimental foundation for gaining further insights into the mechanisms of action within tumor cells.

## 2. Results

### 2.1. Establishment of A549, GFP-OE, and P2RY12-OE Cell Lines

**Figure 1:**
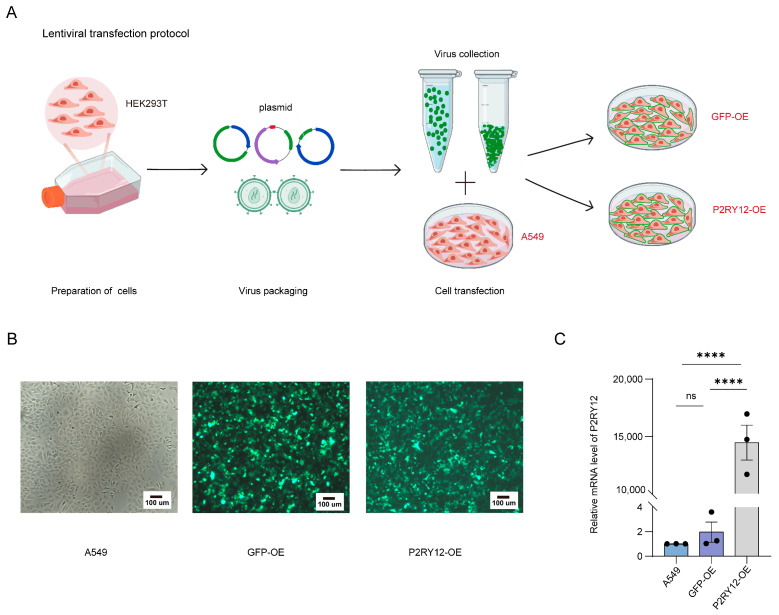
Validation of GFP-OE and *P2RY12*-OE: (**A**) a pictorial representation illustrating the process of plasmid transfection aimed at creating GFP-OE and *P2RY12*-OE; (**B**) the fluorescence of A549, GFP-OE, and *P2RY12*-OE; and (**C**) a histogram illustrating the qRT-PCR results for samples that either were within the normal range or exhibited increased levels of *P2RY12*. Each sample was tested with three biological replicates. ****: *p*< 0.0001, ns means not significant, one-way ANOVA test.

To ensure simplicity and clarity, we hereinafter refer to the original A549 cell line as A549, the cell line that overexpresses GFP as GFP-OE, and the cell line that overexpresses both GFP and *P2RY12* as *P2RY12*-OE (Figure 1A). To confirm the establishment of stable cell lines for GFP-OE and *P2RY12*-OE, the fluorescence of GFP-OE and *P2RY12*-OE was examined using a microscope, while the levels of *P2RY12* mRNA in A549, GFP-OE, and *P2RY12*-OE were assessed using qRT-PCR [18]. Enhanced green fluorescence was observed in both GFP-OE and *P2RY12*-OE (Figure 1B), and the mRNA concentration of *P2RY12* was significantly elevated in the *P2RY12*-OE group relative to A549 and GFP-OE (*p* < 0.0001, one-way ANOVA test, Figure 1C). The findings demonstrated that the creation of GFP-OE and *P2RY12*-OE was accomplished effectively.

### 2.2. Gene Expression Differences Between A549, GFP-OE, and P2RY12-OE

**Figure 2:**
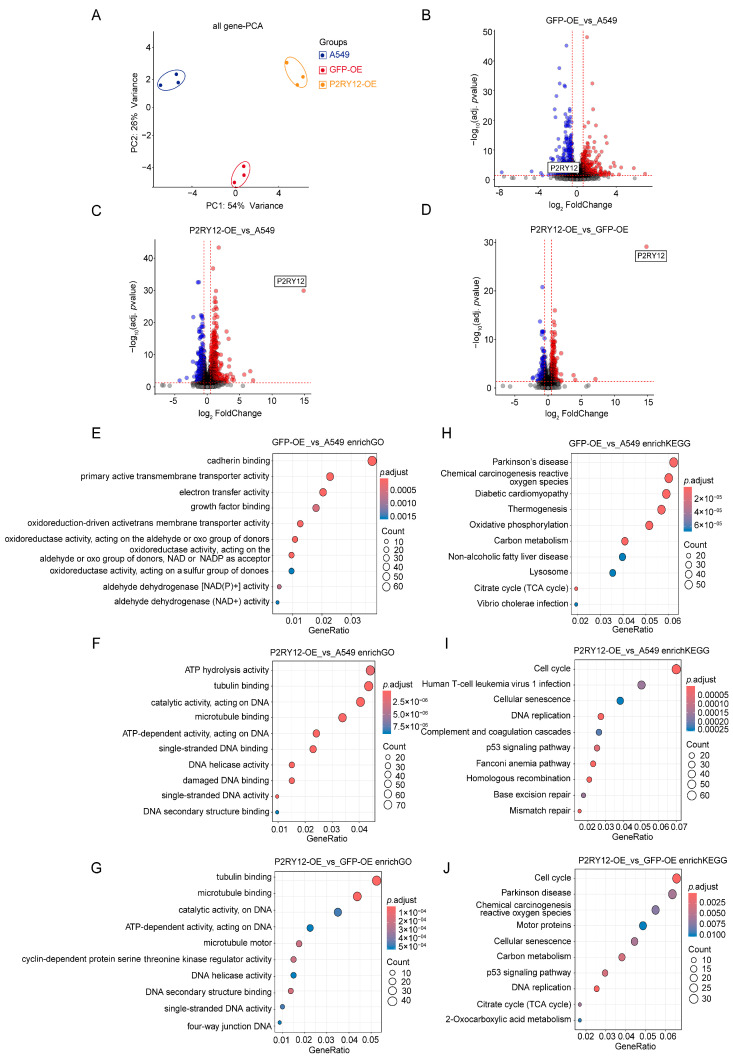
Translational differences between A549, GFP-OE, and *P2RY12*-OE: (**A**) principal component analysis (PCA) based on TPM values of three biological replicates in each group; volcano plots of the DEGs from the comparisons between GFP-OE and A549 (**B**), P2RY12-OE and A549 (**C**), and *P2RY12*-OE and GFP-OE (**D**), Blue dots show genes with down-regulated expression and red dots show genes with up-regulated expression; the top ten enriched GO terms of DEGs from the comparisons between GFP-OE and A549 (**E**), *P2RY12*-OE and A549 (**F**), and *P2RY12*-OE and GFP-OE (**G**); and the top ten enriched KEGG pathways of DEGs from the comparisons between GFP-OE and A549 (**H**), *P2RY12*-OE and A549 (**I**), and *P2RY12*-OE and GFP-OE (**J**).

We then examined the distinctions in expression changes across A549, GFP-OE, and *P2RY12*-OE, with each group having three biological replicates. Initially, the principal component analysis (PCA) of the RNA-seq data, which utilized the TPM values for every gene [19], revealed that the replicates of A549, GFP-OE, and *P2RY12*-OE formed separate clusters (Figure 2A). This finding suggested that there were clear variations among the three groups. Subsequently, examinations of differentially expressed genes (DEGs) (Table S2 [20]) were carried out between GFP-OE and A549 (Figure 2B), *P2RY12*-OE and A549 (Figure 2C), and *P2RY12*-OE and GFP-OE (Figure 2D). Compared with A549, more than 1000 genes were differentially expressed in both GFP-OE (803 genes up-regulated and 994 genes down-regulated, Figure 2B) and *P2RY12*-OE (893 genes up-regulated and 878 genes down-regulated, Figure 2C), while less than 1000 genes were differentially expressed between *P2RY12*-OE and GFP-OE (455 genes up-regulated and 411 genes down-regulated, Figure 2D). As anticipated, the level of *P2RY12* expression was markedly increased in *P2RY12*-OE, establishing this gene as one that is notably up-regulated in contrast to A549 or GFP-OE (Figure 2B–D). To explore the potential biological implications of these DEGs, we executed enrichment analyses for Gene Ontology (GO) and KEGG pathways [21,22] (Figure 2E–J, Figures S1 and S2). The top ten GO molecular function terms identified as being enriched by DEGs in the comparison of GFP-OE and A549 were related to cadherin-binding, electron transfer, growth factor-binding, and oxidoreduction-driven activities (Figure 2E). On the other hand, the differentially expressed genes in *P2RY12*-OE against A549 and *P2RY12*-OE against GFP-OE were chiefly associated with tubulin binding, ATP activity, cyclin-dependent kinases, and DNA structure (Figure 2F,G). Among the top 10 KEGG pathways enriched with DEGs, those from the GFP-OE versus A549 comparison were notably associated with activities involving reactive oxygen species, oxidative phosphorylation, carbon metabolism, and the citric acid cycle (Figure 2H). The analysis of *P2RY12*-OE against A549 and GFP-OE showed that the DEGs were significantly concentrated in pathways pertaining to cell cycle, cellular senescence, and DNA replication, and p53 signaling pathways associated with cell proliferation and migration [23] (Figure 2I,J).

### 2.3. Overlap and Validation of DEGs Between A549, GFP-OE, and P2RY12-OE

**Figure 3:**
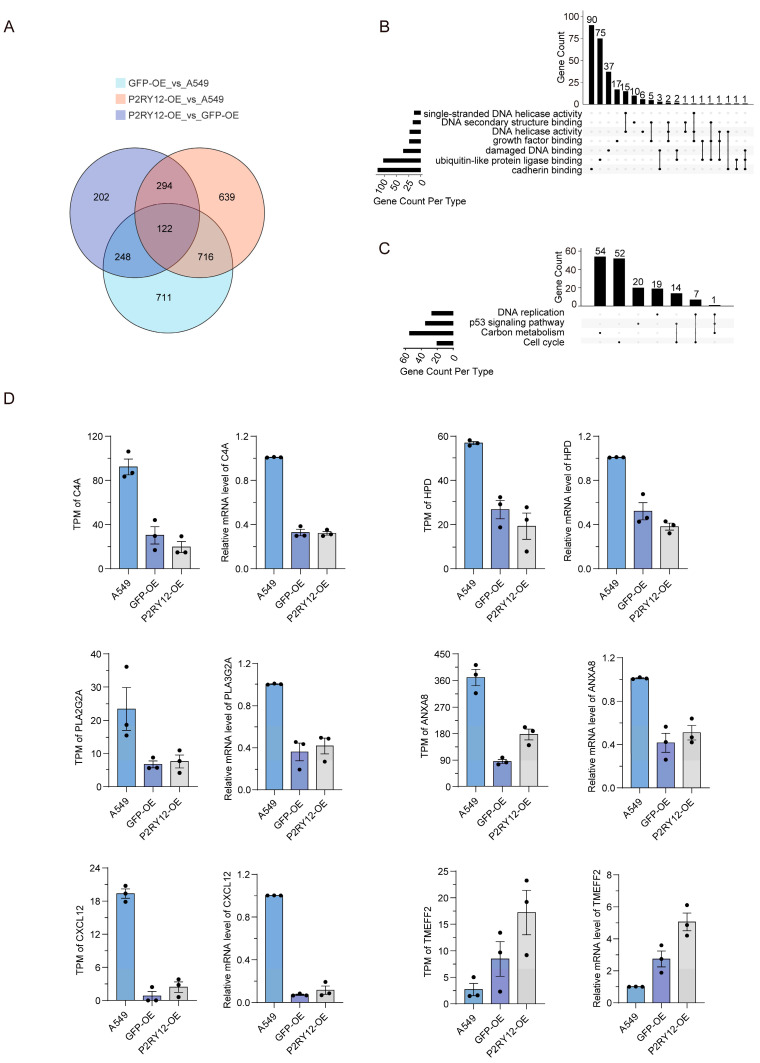
Common DEGs and qRT-PCR validation: (**A**) a Venn diagram of shared DEGs in three comparisons; (**B**) an UpSet diagram showing the intersection of molecular function in three comparisons, the dots with lines show genes were simultaneously enriched in multiple molecular functions; (**C**) an UpSet diagram showing the intersection of KEGG pathways in three comparisons, the dots with lines show genes were enriched across different pathways; and (**D**) bar plots showing the TPM values and qRT-PCR results for six selected DEGs. All experiments were performed in triplicate.

Next, we examined the common DEGs shared in the comparisons. We found 122 genes that displayed changes in expression across all three comparisons, 248 genes that were differentially expressed in both the GFP-OE against A549 and the *P2RY12*-OE against GFP-OE comparisons, and 294 genes that were differentially expressed in both the *P2RY12*-OE against A549 and the *P2RY12*-OE against GFP-OE comparisons (Figure 3A). We conducted an intersection analysis of Gene Ontology (GO) and KEGG pathways between A549, GFP-OE, and *P2RY12*-OE (Figure S3, Tables S3 and S4). Among the seven co-enriched molecular functions, cadherin binding emerged as the most enriched category, accounting for 33.33% of the genes [24]. In contrast, DNA helicase activity was the least enriched, with only 0.02% of the genes [25]. Notably, 12.96% of the genes were simultaneously enriched in multiple molecular functions (Figure 3B). In the KEGG pathway analysis, the cell cycle pathway exhibited the highest degree of enrichment [26], capturing 32.34% of the genes, while DNA replication was the least enriched [27], accounting for 11.38% of the genes. Additionally, 13.17% of the genes were found to be enriched across different pathways (Figure 3C). We proceeded to select several DEGs that potentially play a role in lung cancer progression for additional validation using qRT-PCR. In total, six DEGs were chosen, namely *C4A*, *TMEFF2*, *ANXA8*, *HPD*, *PLA2G2A*, and *CXCL12*. The analysis demonstrated a trend that was consistent with the RNA-seq findings (Figure 3D).

### 2.4. Identification of Alternative Splicing Events in GFP-OE and P2RY12-OE

**Figure 4:**
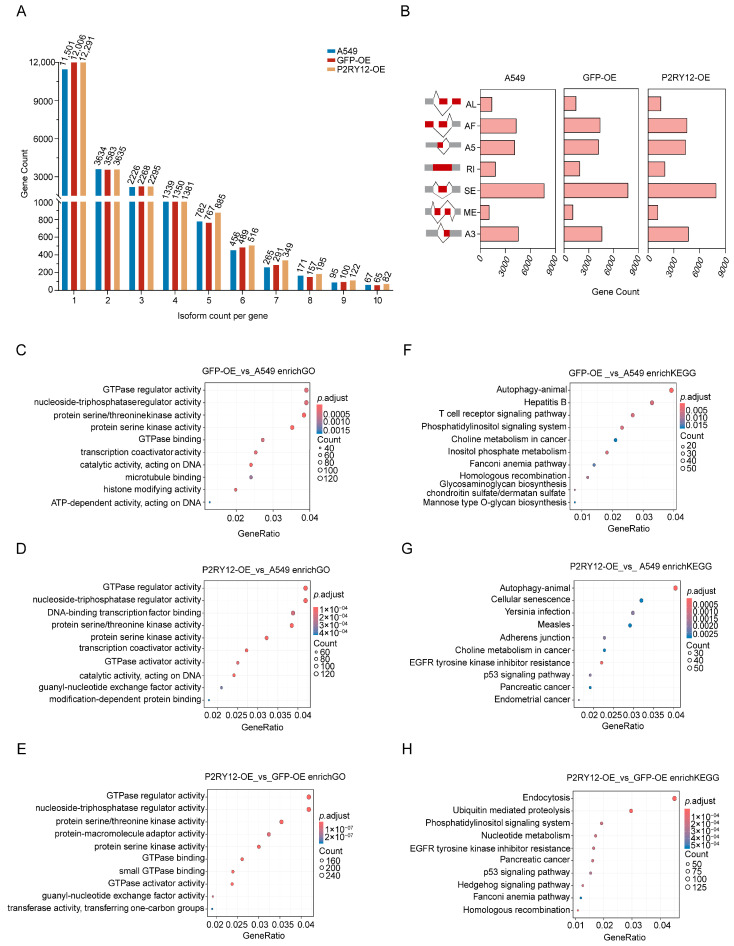
Alternative splicing events in GFP-OE and *P2RY12*-OE: (**A**) the number of genes with different isoform counts; (**B**) the number of genes with different types of alternative splicing events. AL, alternative last exon; AF, alternative first exon; A5, alternative 5′ splice site; RI, retained intron; SE, skipping exon; ME, mutually exclusive exons; A3, alternative 3′ splice site, the grey boxes show constitutive exon, the red boxes show alternatively splice exon; the top ten enriched GO terms of gDTUs from the comparisons between GFP-OE and A549 (**C**), *P2RY12*-OE and A549 (**D**), and *P2RY12*-OE and GFP-OE (**E**); and the top ten enriched KEGG pathways of gDTUs from the comparisons between GFP-OE and A549 (**F**), *P2RY12*-OE and A549 (**G**), and *P2RY12*-OE and GFP-OE (**H**).

Eukaryotes frequently exhibit alternative splicing, a mechanism that influences gene expression after transcription by employing diverse splice sites [28]. This mechanism of transcriptional regulation plays a vital role in the variety of gene functions, enhancing the capabilities of genes and contributing to the intricate protein diversity found within genomes [29]. Hence, exploring the alternative splicing variations between distinct cell lines is a meaningful way to understand the differences that exist among them. Utilizing the Suppa pipeline [30], we conducted an in-depth analysis and categorization of alternative splicing results, as well as a count of various splicing occurrences. In comparison to A549, there was a slight rise in the frequency of isoform formation in both GFP-OE and *P2RY12*-OE. A total of 21,300 genes formed isoforms in GFP-OE, with 12,006 genes showing a single type of isoform and 9294 genes displaying multiple types, which is 43.63% of the overall gene count. In *P2RY12*-OE, 21,993 genes were found to be isoform-forming, where 12,291 genes underwent a single type of isoform, and 9702 genes displayed two or more types of isoforms, accounting for 44.11% of the total number (Figure 4A). An examination of the various types of alternative splicing showed that skipping exon (SE) were the most prevalent, while mutually exclusive exons (ME) occurred the least frequently (Figure 4B and Figure S4). An analysis of GO functional enrichment indicated that the genes with differential transcript usage (gDTUs) [31] related to GFP-OE and *P2RY12*-OE were notably enriched in groups including transcription translation, GTPase regulation, NTPase regulation, protein kinase activity regulation, and ATP action (Figure 4C–E and Figure S5, Table S5). Among the KEGG pathways that showed enrichment in these gDTUs were those involved in autophagy associated with cell proliferation and apoptosis, the T-cell receptor signaling pathway [32], the p53 signaling pathway [33], resistance to EGFR-TKIs, the phosphatidylinositol signaling system [34], and cellular senescence (Figure 4F–H and Figure S6, Table S6).

### 2.5. Overlap Between DEGs and gDTUs

**Figure 5:**
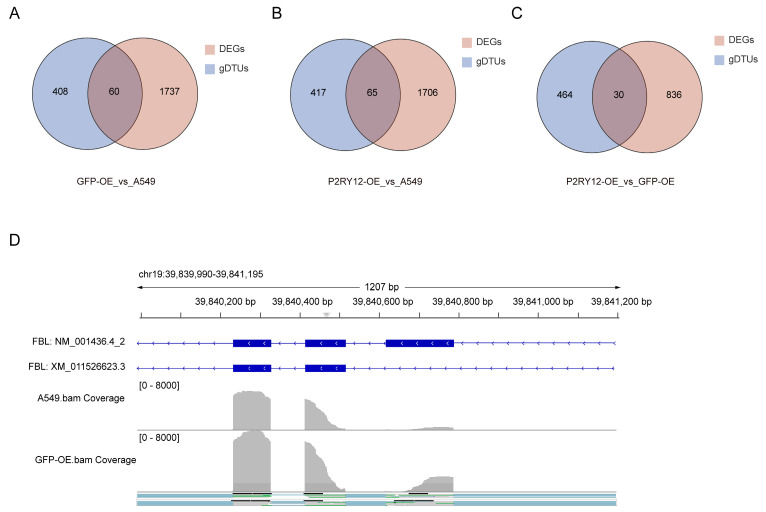
Overlap between DEGs and gDTUs. Venn plots depicting the intersection of DEGs and gDTUs in the comparison between GFP–OE against A549 (**A**), *P2RY12*–OE against A549 (**B**), and *P2RY12*–OE against GFP–OE (**C**); and (**D**) the visualization of *FBL* expression alterations in GFP-OE compared to A549 using IGV.

Lastly, we examined the possible associations between DEGs and gDTUs. The analysis revealed that there were 60, 65, and 30 genes that overlapped among DEGs and gDTUs when comparing GFP-OE to A549, *P2RY12*-OE to A549, and *P2RY12*-OE to GFP-OE, respectively (Figure 5A–C, Table S7). The *FBL* (fibrillarin) gene served as a case study for both DEG and gDTU (Figure 5D). The corresponding protein fibrillarin is a vital component of a nucleolar small nuclear ribonucleoprotein (snRNP) particle, which is thought to be involved in the initial processing of pre-ribosomal RNA [35]. Based on the GTEx data, this gene is highly expressed in various tissues, including the cervix and ovary [36], and it has a pair of isoforms, with the second exon of NM_001436.4_2, spanning 171 bp, being left out in XM_011526623.3 (Figure 5D). In A549, the estimated TPM value of NM_001436.4_2 was 190.5, while that of XM_011526623.3 was 263.1. In GFP-OE, the estimated TPM value of NM_001436.4_2 was 406.3, while that of XM_011526623.3 was 171.5. The delta-PSI measurement for NM_001436.4_2 was determined to be 0.27, accompanied by an estimated empirical *p*-value of 0.0015 (Table S8). Due to the relatively stable expression of XM_011526623.3 in A549 cells and the more than twofold rise in NM_001436.4_2, the *FBL* gene was classified as differentially expressed (adjusted *p*-value = 0.019). In accordance with TPM values, there was a distinct increase in sequencing depth observed in the second, third, and fourth exons, with the second exon presenting the most pronounced elevation. Together, the findings from the *FBL* case indicated that a gene that shows different levels of expression could also be associated with distinct transcript usage, hinting at the potential impact of alternative splicing on the control of gene expression [37].

## 3. Discussion

The process of introducing plasmids into cells is commonly employed in research focused on gene function, with factors such as the size of plasmid segments, their abundance, the type of vector used, and sequences influencing the efficiency of cellular transfection [38,39]. These influences control the various processes of gene transcription and translation, resulting in diverse transcription levels and alternative splicing variations [40]. This study investigated the effects of transfecting the *P2RY12* target gene plasmid and the empty GFP plasmid to determine whether they would cause variations in transcription within A549 cells. Notable changes in gene expression and alternative splicing were detected due to the overexpression of GFP and *P2RY12*. In addition, we found that the heightened expression of GFP and *P2RY12* might affect the regulation of genes related to the growth of cancer cells. The data suggest that the increased expression of GFP and *P2RY12* contributes to the regulation of transcriptional and post-transcriptional activities in the A549 cell line.

Acting as a connector for the flow of biological information from DNA to proteins, mRNA plays a vital role, while the collective identity of all expressed genes and their transcript levels is termed the transcriptome [41]. The regulation of gene transcription by cells allows them to respond effectively to signals from within and outside the cell, shedding light on patterns of gene expression, different splice isoforms, and the management of transcription factors [42,43]. This knowledge can assist in recognizing shifts in specific gene expressions and in anticipating gene functions, thus elucidating their involvement in various biological processes. The use of RNA-sequencing technology offers extensive insights into transcripts and enables the precise examination of variations in gene expression, the identification of novel transcripts, and the accurate detection of alternative splicing events, which are crucial to understanding the regulation of gene expression [44]. One significant yet challenging aspect of RNA transcription is alternative splicing (AS), a post-transcriptional mechanism that allows a single gene to generate various mature mRNA isoforms, leading to the production of different proteins [45,46]. The main types of cellular alternative splicing include alternative last exon (AL), alternative first exon (AF), alternative 5′ splice site (A5), retained intron (RI), exon skipping (ES), mutually exclusive exons (ME), and alternative 3′ splice site (A3). In humans, ES stands out as the most frequently observed AS event [47,48]. Research from extensive genomic studies suggests that approximately 95% of human genes are influenced by alternative splicing [49]. A wealth of research points to the undeniable influence of alternative splicing in the onset of several cancers, including LUAD (lung adenocarcinoma), as it is markedly disrupted in cancer and plays a role in many attributes of tumor cells [50]. Alternative splicing activities in lung cancer have implications for biological processes that govern tumor proliferation [51]; consequently, studying the regulatory mechanisms tied to AS will broaden our knowledge of lung cancer.

It was previously reported that transfected plasmid DNA was doped into the nucleus during end-stage nuclear envelope reorganization, which may affect the gene expression and biological traits of the cells [52]. The reporter gene GFP and the purinergic receptor *P2RY12* are both activated by the transcriptional processes of the host cell in response to lentiviral vectors, leading to the expression of specific target genes [53]. The pLenti is a lentiviral vector, and the incorporation of the *P2RY12* sequence results in a longer plasmid, which might alter transfection efficiencies and subsequently affect gene expression levels [54]. GFP is commonly used as a control vector and for tagging target genes, showing little effect on cell growth. Nonetheless, some studies suggest that GFP expression may increase cellular burden and potentially induce off-target effects at the transcriptional level [55,56]. The biosynthesis of GFP inevitably induces oxidative stress within cells. This oxidative stress can damage cellular components, including lipids, proteins, and DNA, thereby disrupting numerous biological pathways [57]. Persistent overexpression of GFP may further strain intracellular resources by occupying ribosomes and translational machinery. Additionally, GFP mRNA or protein may interfere with host RNA metabolism through interactions with RNA-binding proteins (e.g., microRNAs or RBPs) or by binding to other host transcription factors (e.g., NF-kB or Sp1). This competition for resources could consequently affect the transcriptional and translational processes of other genes [58]. In a bacterial study, it was proposed that transcription-induced supercoiling could uncouple H-NS-mediated silencing, suggesting that transcription-induced anti-silencing does not require transcription to reach the silenced gene and can function over a distance [59]. Drawing from this concept, we hypothesize that the location of GFP insertion, which may result in local DNA structural changes or influence the regulatory elements within the plasmid, could disrupt or activate neighboring genes. Additionally, the chemicals or methods applied in transfection may have a secondary effect on apoptosis and the cell cycle [60], highlighting the need for further studies to verify these predictions. To substantiate our results, it is necessary to integrate other cancer cell lines into the analysis.

An increase in the expression of *P2RY12*, a member of the G protein-coupled receptor family, hinders the proliferation and migration of A549 cells [10], which is of clinical importance in the realm of lung cancer, particularly lung adenocarcinoma (LUAD). Studies have shown that *P2RY12* is associated with M2-type macrophage and dendritic cell infiltration, which may affect the tumor microenvironment and cellular activities such as proliferation and migration [61]. We discovered in this analysis that *P2RY12* has a substantial impact on the expression levels of several genes related to cell division and migration, notably *C4A*, *TMEFF2*, *ANXA8*, *HPD*, *PLA2G2A*, and *CXCL12*. The gene *C4A* encodes for the acidic type of complement factor 4, which plays a role in the complement system and is released from complement component C4 upon activation [62]. By attaching to the PAR4 receptor, it can potentially affect lung cancer cell invasion and metastasis, thereby acting on cancer cells [63]. ANXA8, a protein from the Annexin family that associates with cell membranes, regulates the proliferation and migration of lung cancer cells through its effect on the EGFR-AKT-mTOR signaling pathway [64]. Known as stromal cell-derived factor-1 (SDF-1), CXCL12 is a small protein within the CXC chemokine family [65]. Investigations have revealed that the presence of CXCL12 and its receptor CXCR4 in non-small cell lung cancer correlates with tumor development, infiltration, and metastasis [66]. These genes are closely related to the proliferation and migration of lung cancer A549 cells, and their downstream targets are regulated by *P2RY12*, which implies that *P2RY12* may affect the progression of non-small cell lung cancer by regulating the expression of these genes. Additional research is required to establish and verify the connection between *P2RY12* and the associated genes.

It was observed that the DEGs and gDTUs regulated by *P2RY12* showed a certain level of enrichment in the pathways linked to p53 signaling, apoptosis, and the cell cycle. The pathway associated with p53 is significant in modulating the process of apoptosis within cancer cells. According to recent findings, the use of triple therapy involving aspirin, afatinib, and vincristine has been shown to promote apoptosis in NSCLC cells by stimulating the p53-related signaling mechanisms [67,68]. The p53 pathway is influenced by alternative splicing through its effect on the p53 repressor’s expression, leading to changes in cell division, death, and senescence [69]. Although p53 levels tend to be higher in nearly all cases, the excessive expression of the RNA-binding protein RBM10, for example, can initiate p53-related apoptosis in multiple cancer cells [70]. Our research indicates that *P2RY12* might facilitate tumor development by influencing the alternative splicing of genes associated with the p53 signaling pathway in A549 cells. Additional studies are required to reveal how *P2RY12* influences the regulation of p53.

## 4. Materials and Methods

### 4.1. Cell Culture

The human lung adenocarcinoma cell line A549 was purchased from Kinlogix (Guangzhou, China) and grown in RPMI1640 medium (Gibco, Waltham, MA, USA) containing 10% fetal bovine serum (Gibco, Waltham, MA, USA) and 100 µg/mL streptomycin and 100 U/mL penicillin (Beyotime, Shanghai, China). The Human Embryonic Kidney 293T Cells were purchased from Procell (Wuhan, China) and grown in DMEM (Gibco, Waltham, MA, USA). The cells were incubated at 37 °C with 5% CO_2_ in an incubator.

### 4.2. Plasmid Transfection

The coding sequence of the human *P2RY12* was cloned and ligated into the lentiviral vector for overexpression of *P2RY12* in the A549 cell line [71]. The HEK293T cells used Lipofectamine 3000 (Invitrogen, Waltham, MA, USA) for viral packaging with a pLenti-*P2RY12-*GFP+Puro-3xFlag (Youbio, Changsha, China) plasmid that overexpressed *P2RY12* or an empty vector according to the manufacturer’s protocol. Subsequently, the A549 cells were transfected with viral fluid.

### 4.3. RNA Extraction and Quantitative Real-Time Polymerase Chain Reaction (qRT-PCR)

Total RNA was extracted from A549 cells, GFP-OE cells, and *P2RY12*-OE cells, using TRIzol reagent (Takara, Kyoto, Japan) according to the manufacturer’s instructions. RNA concentration was measured using a Nano-500 micro-spectrophotometer (ALLSHENG, Hangzhou, China), and RNA quality was assessed using agarose gel electrophoresis. Subsequently, complementary DNA (cDNA) was synthesized using a PrimeScript^TM^ FAST RT reagent kit with gDNA Eraser (Takara, Kyoto, Japan). Real-time polymerase chain reactions (qRT-PCR) were carried out using TB Green Premix Ex Taq^TM^ II (Takara, Kyoto, Japan) on a QuantStudio 5 Real-Time PCR instrument (Thermo Fisher Scientific, Waltham, MA, USA), with three biological replicates for each PCR reaction. The relative gene expression levels in various cells were calculated using the 2^−ΔΔCT^ method, with *GAPDH* serving as the internal control [72]. The specific primer sequences, supplied by Sangon (Shanghai, China), are detailed in Table S1. Data analysis for relative gene expression level in various cells was performed utilizing GraphPad Prism 10.0 software. One-way analysis of variance (ANOVA) was applied for comparison between multiple groups. The differences between groups were considered statistically significant when the two-side *p* < 0.05.

### 4.4. RNA-Seq Data Analysis and Validation

RNA sequencing was carried out on the MGI platform, where the clean reads in fastq format were quantified at the transcript level using salmon (v0.14.1, [73], options: -l ISF --gcBias). The transcript level quantification results were converted to gene level by tximport (v1.30.0, [31]). Genes that showed significant expression differences were determined by applying DEseq2 (v1.42.0, [74]), with an adjusted *p*-value below 0.05 as the threshold. The Gene Ontology (GO) and Kyoto Encyclopedia of Genes and Genomes (KEGG) [22] enrichment of differentially expressed genes (DEGs) were performed using clusterProfiler with default parameters (v4.10.1, [75]). Furthermore, we validated several DEGs identified from the RNA sequencing analysis by conducting qRT-PCR experiments, using primers specifically created for each respective gene (Table S1). All DEGs’ expression levels were standardized against the internal control gene *GAPDH*.

### 4.5. Identification and Quantification of Alternative Splicing Events

Alternative splicing events were identified and quantified through the application of the Suppa pipeline (v2.3, [30]). The fasta version of the human genome GRCh38.p14 was retrieved from UCSC (accessed in December 2023), while the associated gtf format annotation file was downloaded from UCSC (https://hgdownload.soe.ucsc.edu/goldenPath/hg38/bigZips/genes/hg38.ncbiRefSeq.gtf.gz, accessed on 20 December 2023). The Y chromosome and patch chromosome were removed in this research. The creation of ioi and ioe files, which extracted the relevant transcript and exon information from the annotation, was carried out via the generateEvents module (options: -e SE SS MX RI FL), utilizing the gtf format annotation as the source file. The calculation of percent spliced in (PSI) values for each event or transcript was performed using the psiPerEvent and psiPerIsoform modules, respectively. Utilizing the diffSplice module with the settings -m empirical and -gc, the analysis of differential transcript usage (DTU) was carried out, which not only determined the delta PSI values for transcripts between two groups but also yielded an empirical *p*-value for each transcript. An in-house script was employed to sift through the final outcomes, ensuring that only alternative splicing events present in a minimum of two out of three biological replicates were kept. The visualization of structural information and RNA sequencing data for focal genes was carried out using the Integrative Genomics Viewer (IGV 2.18.4, [76]).

## 5. Conclusions

In essence, our research confirms that the transfection of GFP and P2RY12 plasmids has a substantial impact on gene expression in cells, as demonstrated with RNA-seq technology, while also uncovering their role in regulating alternative splicing. This research not only confirms that the introduction of plasmids can lead to considerable alterations in gene expression but also highlights their ability to regulate processes after transcription has occurred. Investigating these transformations in detail allowed us to better grasp the complex biological interactions involved in plasmid transfection, especially how they influence gene transcription and the regulation of gene expression at the RNA level. These results play a role in refining strategies for gene transfection, which can lead to greater efficiency and safety in gene therapy. Moreover, they pave the way for novel research directions to examine the effects of plasmid transfection on the functions of cells. Our findings suggest that *P2RY12* may affect cancer progression in A549 cells by inhibiting the activity of genes linked to cell division and migration. Our findings also emphasize that further studies of *P2RY12*-regulated alternative splicing will contribute to an accurate understanding of the signaling network that directs carcinogenesis, as well as the potential for *P2RY12*-targeted therapies.

## Data Availability

The data presented in this study are openly available from the National Genomics Data Center at https://ngdc.cncb.ac.cn/search/all?q=PRJCA036439 (accessed on 21 February 2024), reference numberPRJCA036439.

## Supplementary Materials

The following supporting information can be downloaded at https://www.mdpi.com/article/10.3390/ijms26072973/s1.

## Abbreviations

The following abbreviations are used in this manuscript:

DEGs	Differentially expressed genes
gDTUs	Genes with differential transcript usage
GFP-OE	GFP over-expression
*P2RY12*-OE	*P2RY12* over-expression
AS	Alternative splicing
AL	Alternative last exon
AF	Alternative first exon
A5	Alternative 5′ splice site
RI	Retained intron
ES	Exon skipping
ME	Mutually exclusive exons
A3	Alternative 3′ splice site
LUAD	Lung adenocarcinoma
PCA	Principal component analysis
GO	Gene Ontology
*FBL*	Fibrillarin
*C4A*	Complement C4A
*TMEFF2*	Transmembrane protein with an EGF-like and two Follistatin-like domains 2
*ANXA8*	Annexin A8
*HPD*	4-hydroxyphenylpyruvate dioxygenase
*PLA2G2A*	Phospholipase A2, Group IIA
*CXCL12*	C-X-C motif chemokine ligand 12
NSCLC	Non-small cell lung cancer
qRT-PCR	Quantitative real-time polymerase chain reaction
