# Supplementary material for: Alterations in Gene Expression and Alternative Splicing Induced by Plasmid-Mediated Overexpression of GFP and P2RY12 Within the A549 Cell Line
Source: Int J Mol Sci. 2025 Mar 25;26(7):2973. doi: 10.3390/ijms26072973 (PMC11988474; doi:10.3390/ijms26072973)
Supplement: Supplementary file 1 [file ijms-26-02973-s001.zip › ijms-3517450- Supplementary Materials.pdf]

# Supplementary Materials

## Supplementary figures

Figure. S 1 Top fifty enriched GO term of DEGs. .......... 2
Figure. S 2 Top fifty enriched KEGG of DEGs. .......... 3
Figure. S 3 Venn plots of GO and KEGG intersect. .......... 4
Figure. S 4 Probability of AS type. .......... 4
Figure. S 5 Top fifty enriched GO term of gDTUs. .......... 5
Figure. S 6 Top fifty enriched KEGG of gDTUs. .......... 6

## Supplementary tables

Table. S 1 Primer sequences .......... 7
Table. S 2 The details of the DEGs .......... 7
Table. S 3 GO analysis of DEGs .......... 7
Table. S 4 KEGG analysis of DEGs .......... 7
Table. S 5 GO analysis of gDTUs .......... 7
Table. S 6 KEGG analysis of gDTUs .......... 7
Table. S 7 Overlap of DEGs and gDTUs .......... 7
Table. S 8 The dPSI-TPM of FBL .......... 7

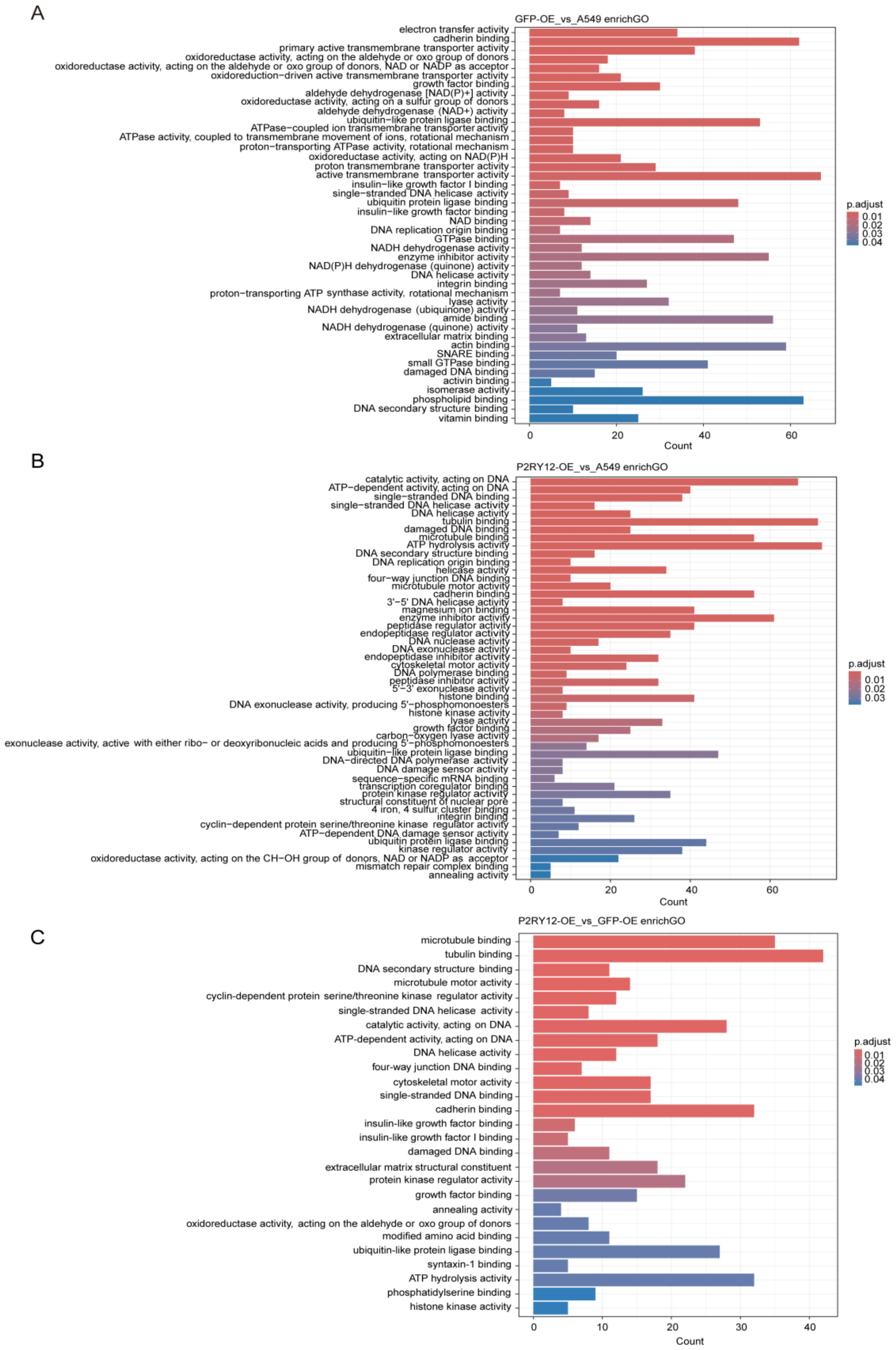


**Figure. S 1** Top fifty enriched GO term of DEGs.
The top fifty enriched GO term of DEGs from the comparisons between GFP-OE and A549 (A), P2RY12-OE and A549 (B), P2RY12-OE and GFP-OE (C).

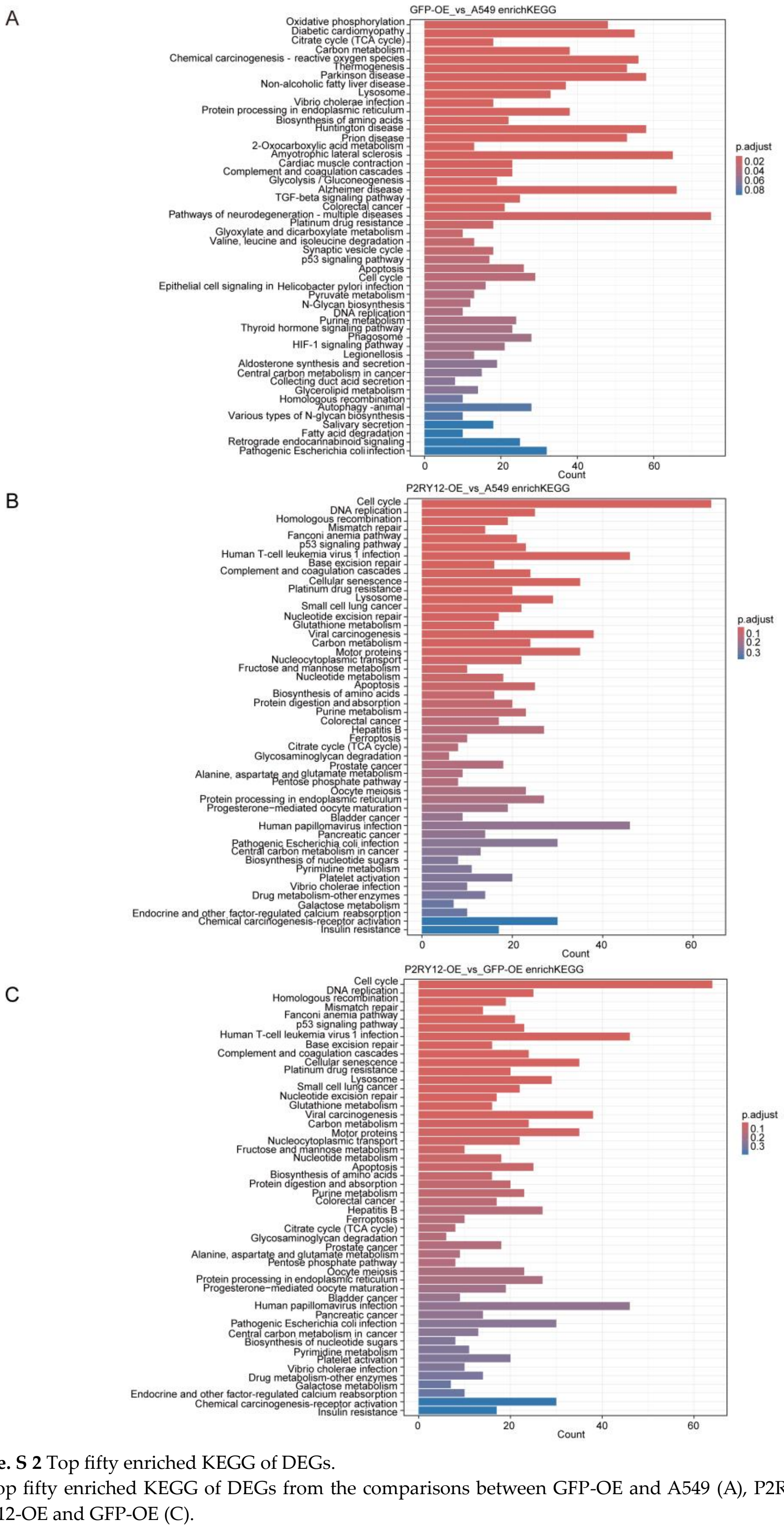


**Figure. S 2** Top fifty enriched KEGG of DEGs.

The top fifty enriched KEGG of DEGs from the comparisons between GFP-OE and A549 (A), P2RY12-OE and A549 (B), P2RY12-OE and GFP-OE (C).

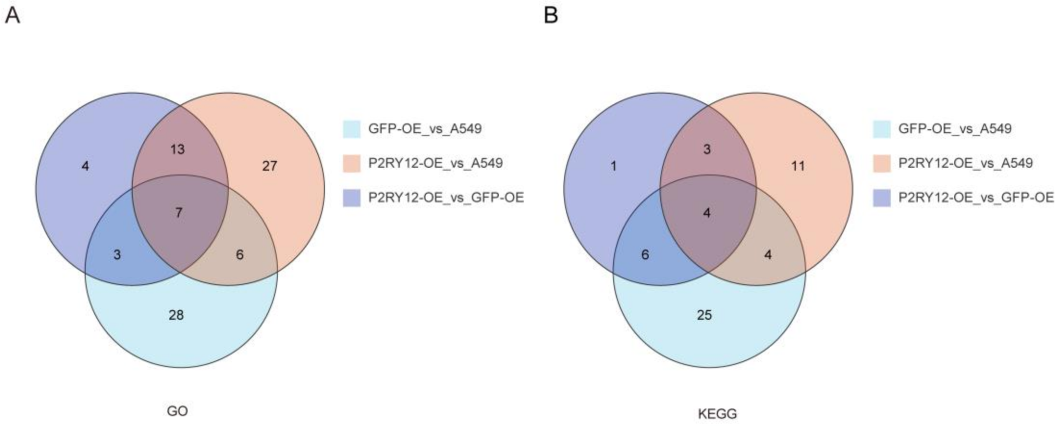


**Figure. S 3** Venn plots of GO and KEGG intersect.
(A), Venn plots depicted the intersection of GO term in the comparison between GFP-OE against A549, P2RY12-OE against A549, and P2RY12-OE against GFP-OE; (B), Venn plots depicted the intersection of KEGG pathway in the comparison between GFP-OE against A549, P2RY12-OE against A549, and P2RY12-OE against GFP-OE.

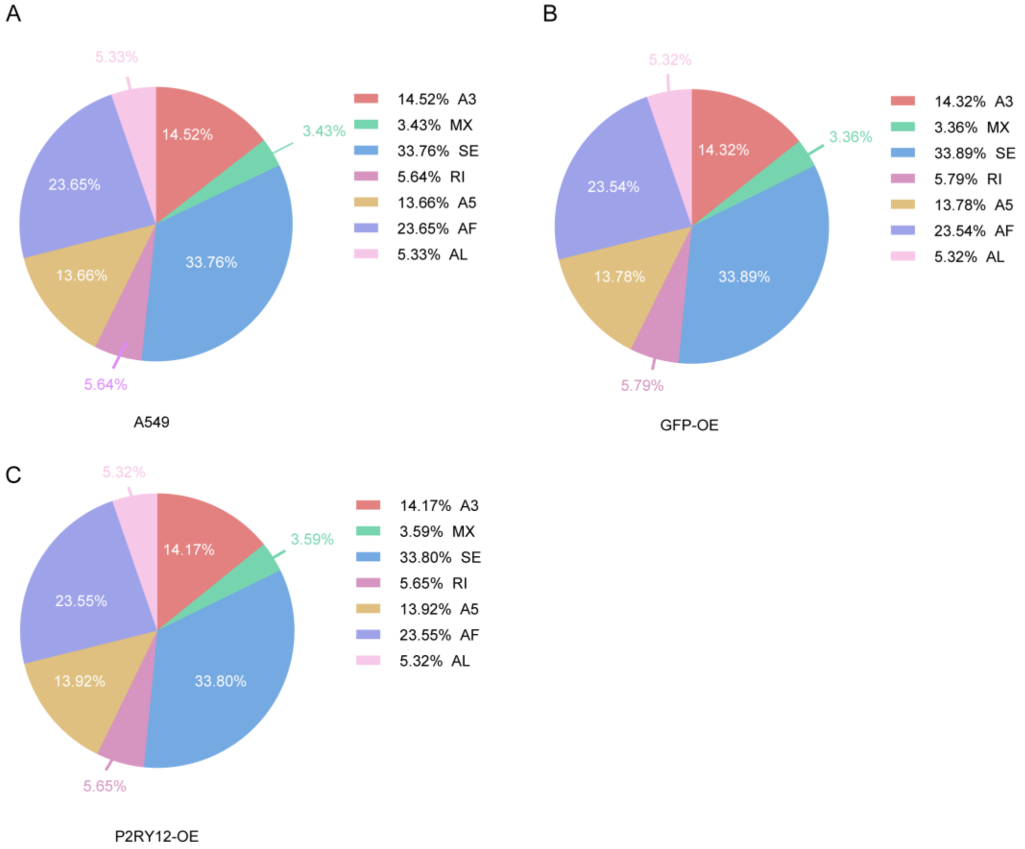


**Figure. S 4** Probability of AS type.
(A), The probability of AS type in A549; (B), The probability of AS type in GFP-OE; (C), The probability of AS type in P2RY12-OE.

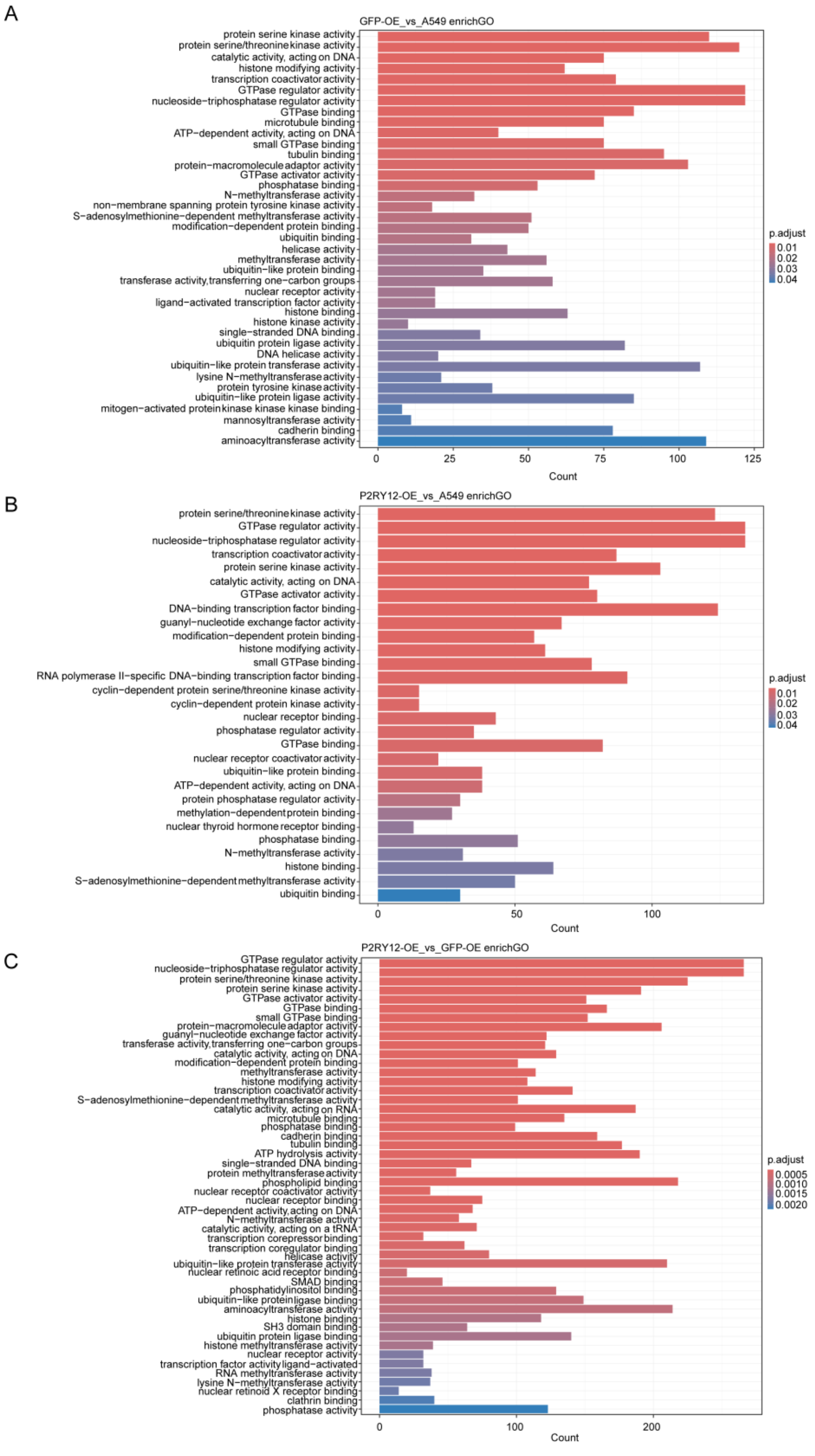


**Figure. S 5** Top fifty enriched GO term of gDTUs.

The top fifty enriched GO term of gDTUs from the comparisons between GFP-OE and A549 (A), P2RY12-OE and A549 (B), P2RY12-OE and GFP-OE (C).

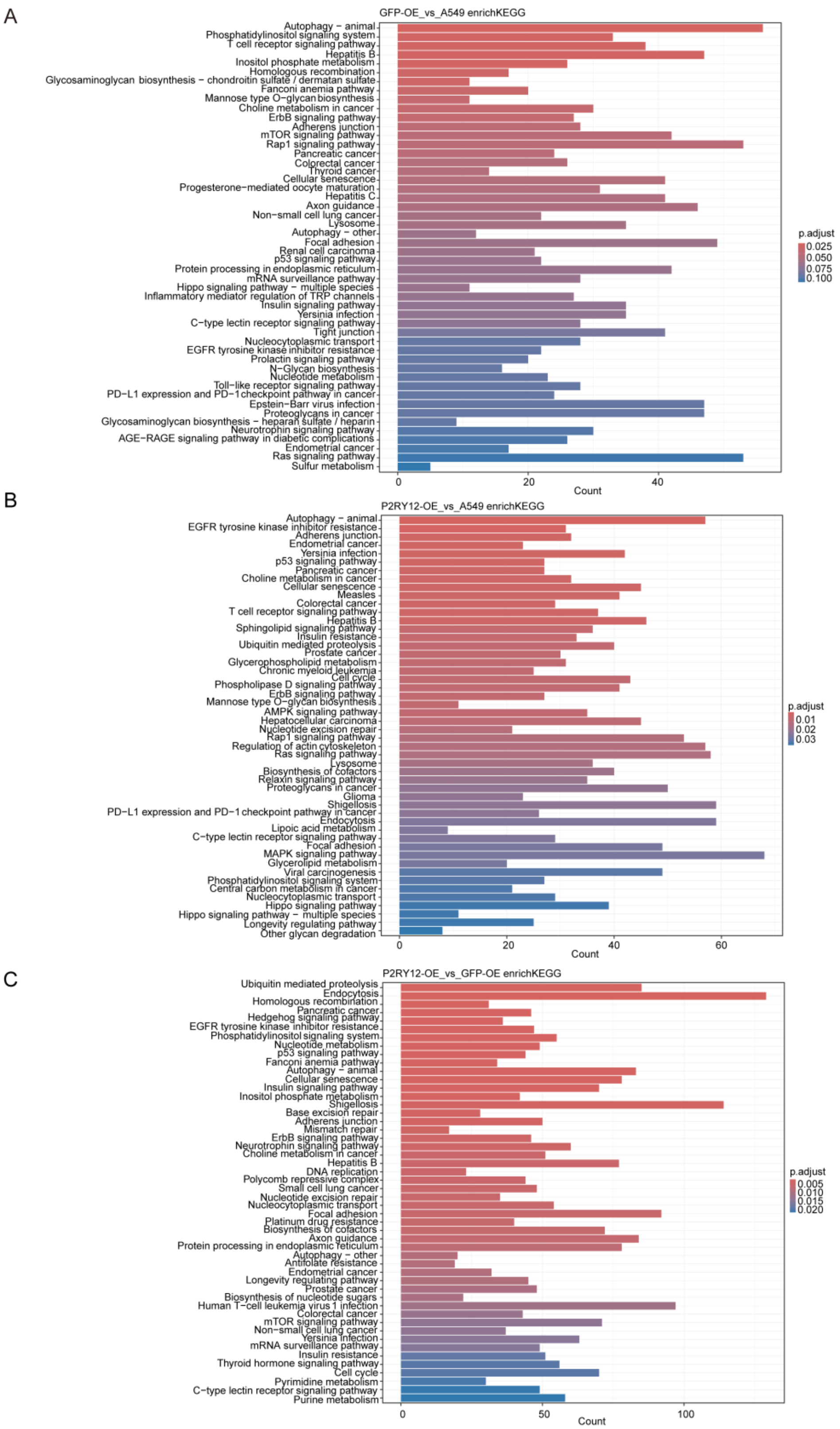


**Figure. S 6** Top fifty enriched KEGG of gDTUs.

The top fifty enriched KEGG of gDTUs from the comparisons between GFP-OE and A549 (A), P2RY12-OE and A549 (B), P2RY12-OE and GFP-OE (C).

**Table. S 1** Primer sequences

The table. S 1 describes the primer sequences of six genes, TMEFF2, ANXA8, HPD, PLA2G2A, C4A, CXCL12, and P2RY12, which were used to perform qRT-PCR experiments.

**Table. S 2** The details of the DEGs

The table. S 2 shows the differentially expressed genes (DEGs) between GFP-OE and A549, P2RY12-OE and A549, as well as P2RY12-OE and GFP-OE.

**Table. S 3** GO analysis of DEGs

The table. S 3 shows the GO molecular function terms of DEGs between GFP-OE and A549, P2RY12-OE and A549, as well as P2RY12-OE and GFP-OE (p.adjust<0.05).

**Table. S 4** KEGG analysis of DEGs

The table. S 4 shows the KEGG pathways analysis of DEGs between GFP-OE and A549, P2RY12-OE and A549, as well as P2RY12-OE and GFP-OE (p.adjust<0.05).

**Table. S 5** GO analysis of gDTUs

The table. S 5 shows the GO molecular function terms of gDTUs between GFP-OE and A549, P2RY12-OE and A549, as well as P2RY12-OE and GFP-OE (p.adjust<0.05).

**Table. S 6** KEGG analysis of gDTUs

The table. S 6 shows the KEGG pathways analysis of gDTUs between GFP-OE and A549, P2RY12-OE and A549, as well as P2RY12-OE and GFP-OE (p.adjust<0.05).

**Table. S 7** Overlap of DEGs and gDTUs

The table. S 7 shows the Overlap of DEGs and gDTUs between GFP-OE and A549, P2RY12-OE and A549, as well as P2RY12-OE and GFP-OE.

**Table. S 8** The dPSI-TPM of FBL

The table. S 8 exhibits the dPSI-TPM of two isoforms NM_001436.4_2 and XM_011526623.3 in the FBL.
